# Archetype analysis and the PHATE algorithm as methods to describe and visualize pregnant women’s levels of physical activity knowledge

**DOI:** 10.1186/s12889-024-18355-7

**Published:** 2024-04-15

**Authors:** Marek Karwański, Urszula Grzybowska, Ewa Mierzejewska, Katarzyna Szamotulska

**Affiliations:** 1 Department of Applied Mathematics, University of Life Sciences-SGGW, Nowoursynowska 159, 02-776 Warsaw, Poland; 2 Department of Epidemiology and Biostatistics, Institute of Mother and Child, Kasprzaka 17a, 01-211 Warsaw, Poland

**Keywords:** Archetypal analysis, PHATE, Knowledge of physical activity in pregnant women, PCA, Patterns of knowledge

## Abstract

The knowledge of physical activity (PA) recommended for pregnant women and practical application of it has positive impact on the outcome. Nevertheless, it is estimated that in high-income countries over 40% of pregnant women are insufficiently physically active. One of the reasons is insufficient knowledge pregnant women have about allowed effort during pregnancy and both recommended and not recommended physical activities. Description of knowledge about physical activity the women have and distinguishing patterns of their knowledge is becoming an increasingly important issue. A common approach to handle survey data that reflect knowledge involves clustering methods or Principal Component Analysis (PCA). Nevertheless, new procedures of data analysis are still being sought. Using survey data collected by the Institute of Mother and Child Archetypal analysis has been applied to detect levels of knowledge reflected by answers given in a questionnaire and to derive patterns of knowledge contained in the data. Next, PHATE (Potential of Heat-diffusion for Affinity-based Trajectory Embedding) algorithm has been used to visualize the results and to get a deeper insight into the data structure. The results were compared with picture derived from PCA. Three archetypes representing three patterns of knowledge have been distinguished and described. The presentation of complex data in a low dimension was obtained with help of PHATE. The formations revealed by PHATE have been successfully described in terms of knowledge levels reflected by the survey. Finally, comparison of PHATE with PCA has been shown. Archetype analysis combined with PHATE provides novel opportunities in examining nonlinear structure of survey data and allows for visualization that captures complex relations in the data. PHATE has made it possible to distinguish sets of objects that have common features but were captured neither by Archetypal analysis nor PCA. Moreover, for our data, PHATE provides an image of data structure which is more detailed than interpretation of PCA.

**Supplementary Information:**

The online version contains supplementary material available at 10.1186/s12889-024-18355-7.

## Introduction

Physical activity during pregnancy has positive impact on its course and the outcome [1]. It is recommended by professional medical bodies that in the absence of specific obstetric or medical complications pregnant women should be encouraged either to continue or to initiate physical activity, with some exceptions regarding its particular kinds and intensity [2, 3]. Nevertheless, it is estimated that in high-income countries physical activity of the majority of women expecting a child does not reach the endorsed level of moderate-intensity exercise for at least 150 min per week [4–6].

Insufficient physical activity of women in normal pregnancy may result from several reasons, such as pregnancy complaints, fear of harming the foetus, lack of time or socio-cultural conditions [7]. Not without importance is also insufficient knowledge of women, about the latest recommendations, which was considered as a theoretical domain in the framework for use in behaviour change [8].

Improper knowledge the women have on this topic goes into two opposite directions. The first one is associated with the traditional perception derived from medical knowledge of several decades ago that exercise in pregnancy is potentially harmful and it may result in practice in “the rest habit” and insufficient activity. The second is based on the lack of knowledge about intensity and particular types of physical activities, which are not recommended in the period of pregnancy and, in practice, it may result in undertaking inadvisable activities.

During last years, there were some trials to measure knowledge regarding physical activity in pregnant women undertaken [9–11], but as a result neither the two opposites of incorrect knowledge were taken into account nor the total score of knowledge suitable for further analyses was developed.

In general, existing methods used to analyse knowledge reflected by answers collected in a survey are not always suitable e.g., when the researcher is interested in detecting patterns of knowledge without reducing the number of questions so that full dimensionality is preserved. Principal Component Analysis (PCA), serves the purpose of dimensionality reduction and data visualization [12–15]. On the other hand, segmentation methods, especially these based on distances (k-means, hierarchical clustering methods), preserve full dimensionality while providing an average representation of the distinguished groups. Another approach to multivariate analysis is Archetypal analysis. Archetypes are extreme points in the observation space. Each observation can be represented as a combination of the distinguished archetypes. Archetypal analysis as a tool for the statistical description of multidimensional objects was introduced by Breiman and Cutler in 1994 [16] and is now used in many fields [17–21]. As far as cluster analysis can be described as a segmentation method, Archetypal analysis corresponds to the search of trend makers, i.e., objects that can be regarded as extreme.

The aim of the research presented in this paper is to describe the knowledge on physical activity during pregnancy, distinguish patterns of knowledge, similarities and dissimilarities in the group of women under consideration.

To reach the goal, we apply Archetype analysis. To visualize the results in low-dimensional space, a dimensionality-reducing algorithm is needed. The PHATE model is used to serve this purpose. Results presented in this paper show that it allows for a better insight into the data structure, while maintaining its complexity, than PCA, traditionally used for data visualization. The analysis was carried out according to the following scheme:

1. Data collection and cleansing;
2. Archetypal model;
3. PHATE and PCA transformation and visualization;
4. Conclusion useful for shaping health attitudes.

The paper is organized as follows. We first describe methods used in the research: Archetypal analysis for pattern recognition, as well as PCA and PHATE for data visualization. In the next section we describe our data and the encoding process. In the subsequent section we present the results. The last part of the paper is devoted to discussion and conclusions.

## Methods

The following analysis is a part of the research conducted by the Institute of Mother and Child. The cohort country-wide study concerned the determinants of healthy behaviours in pregnancy like adequate nutrition and physical activity including proper knowledge. For this purpose, a special set of questionnaires was developed. They were tested in the cross-sectional test survey, which was carried out on a group of 142 pregnant women^1^, 30%, 33% and 37% in I, II and III trimester of pregnancy, respectively, in 6 gynaecology and obstetrics outpatient clinics in Poland, Mazowieckie region, between October and November 2017. Seventeen questions regarding knowledge about physical activity were adapted from a previously published questionnaires [9–11] or newly developed to cover all main aspects of the latest medical recommendations [2, 3, 8]. The questions contained in the survey are added in supplementary information. The study design has been described in detail elsewhere [22].

Each woman was treated as an object described with a vector of values, which were her answers to the questions included in the survey. The answers in the coding process were given numerical values and were treated as points in a metric multidimensional space. The process is described in details in Section “Data and encoding process”. To handle sparse missing data the approximate bayesian bootstrap implementation of hot-deck technique was used. In general, large dispersion of points in multidimensional space makes it difficult to identify groups of similar objects [15]. In such cases cluster analysis models are used as a standard [15]. In this paper, we propose an implementation of another tool - Archetypal analysis [16–18].

### Archetypal analysis as a search for trend makers

We can formulate the problem of archetypes as follows [16–18]. Let us denote by $$ X$$ an $$ n\times m$$ matrix representing a multidimensional data set with $$ n$$ observations and $$ m$$ attributes. The goal of Archetypal analysis is to find a $$ m\times k$$ matrix $$ Z$$ defining archetypes. More precisely, we look for two matrices of $$ \alpha $$ and $$ \beta $$ coefficients of dimension $$ n\times k$$ that minimise the residual sum of squares:

$$ RSS={\left\| {X-\alpha {Z}^{T}} \right\|}_{F}$$

where $$ Z={X}^{T}\beta $$, the optimised parameters $$ {\alpha }_{ij}$$ are weights (coefficients) of convex combinations of archetypes for individual observations: $$ \sum _{j=1 }^{k}{\alpha }_{ij}=1,{\alpha }_{ij}\ge 0$$, $$ i=1,.,n$$, optimised parameters $$ {\beta }_{ij}$$ are weights of archetypes in the space of objects, $$ \sum _{i=1}^{n}{\beta }_{ij}=1, {\beta }_{ij}\ge 0 $$, and$$ \left\| \, \right\|_{F} $$ is the Frobenius matrix norm. Constraints mean that equation above approximates the data with combinations of archetypes, i.e., $$ X=\alpha {Z}^{T}$$, and the archetypes are combinations of weighted data points, i.e., $$ Z={X}^{T}\beta $$. Optimisation also concerns the number of archetypes, the hyper-parameter denoted by $$ k $$[18].

Archetypal analysis maps data points using prototypes, which are themselves convex combinations of data points. Hence, there is some similarity to k-means clustering. However, instead of approximating each data point with a single set of similar points (cluster), Archetypal analysis approximates each data point with a convex combination of a set of prototypes. Using a convex combination causes the observed data points to be a cloud spanning between archetypes. In this sense, archetypes are “pure” and can be interpreted as trend-makers.

Within the framework of Archetypal analysis, we analyse the characteristics of the distinguished archetypes. We can also analyse objects in relation to the received archetypes. We examine to what extent the characteristics of the objects under study coincide with these of archetypes.

### PHATE algorithm for visualization and interpretation

We use PHATE algorithm to present and examine our data. PHATE (Potential of Heat-diffusion for Affinity-based Trajectory Embedding) is an algorithm for visualising the structure of multidimensional data in a low dimensional space [23, 24]. More precisely, the PHATE method is based on the construction of local geometry allowing to understand and present the “shape of the data”. For this purpose, local similarities between the data points are constructed based on the distances determined by the probability distribution, and then the connections between the data points are discovered using the Markov random walk method. In this way, more global relationships are found. PHATE preserves local and global structure in the high-dimensional data, it is not sensitive to noise and presents as much information as possible in low dimensions [23, 24].

The algorithm first computes the distances between the points, which are then transformed by Gaussian kernels into a probability distribution P [23–28]. The advantage of using a Gaussian kernel is to smooth out noisy data and obtain a more stable representation. The use of Markov random walk transitions based on P allows to generate trajectories (graphs) defining geometric structures. In fact, the resulting geometric structures are largely limited to the nearest neighbours of each data point, but also include a global data structure [23, 24]. The distance between points is calculated as

$$ {D}^{diff}\left(x,y\right)\triangleq {\left(\sum _{j}\frac{{\left({p}_{x}\left({y}_{j}\right)-{p}_{y}\left({y}_{j}\right)\right)}^{2}}{{p}^{\infty }\left({x}_{j}\right)}\right)}^{1/2},$$

where ${D}^{diff}$ is a diffusion distance, ${p}_{x}\left( \right)$ denotes probability distribution around point x and ${p}^{{\infty }}\left( \right)$ is a stationary asymptotic distribution defined by Markov process [23, 24]. The diffusion trajectories transform data into Euclidean space, where usual distances describe the relationship between points. In this way, hidden relationships between data points can be described using a simple, intuitive Euclidean distance. Finally, the resulting observation space is reduced e.g., by using non-metric MDS (Multidimensional Scaling) models to achieve low dimensionality [12].

Thus, the PHATE algorithm allows for the construction of a non-linear transformation of multidimensional data, which simultaneously removes noise and preserves the continuous nature of changes in the observation space, enabling data visualization taking into account the real relationships between sampling points. This technique turns out to be quite effective when the data has a globally non-linear character, which is often found as the dominant underlaying pattern in biological systems [23, 24]. Its modification was successfully applied for medical data [29].

### Principal component analysis

Traditionally, Principal Component Analysis (PCA) is used to visualize multidimensional data. It was introduced in 1933 by Hotelling [30]. Its main purpose is to reduce the observation dimensionality, which helps to explore and discover interesting hidden properties in the data. The simplest way to reduce the dimensions is to replace the raw variables with appropriate linear combinations, i.e., weighted averages - projecting the observation space into a linear hyperspace [12–14]:

$$ {w}^{T}X=\sum _{j=1}^{p}{w}_{j}{X}_{j}$$

where $w=({w}_{1},\dots,{w}_{p})$ is a vector of weights defining the projection. This equation is called a standardized linear combination (SLC). In the case of PCA, the weight values are computed based on maximizing the variance in the reduced hyperspace

$$ {PCA}\left(w\right)\triangleq \underset{\left\{w: \left\| {w} \right\|=1\right\}}{\text{arg} \text{max}}Var\left({w}^{T}X\right).$$

The vector $$ w$$ can be found using the spectral decomposition of the covariance matrix $$ Var(\bullet )$$. The values of the variance are then equal to the eigenvalues $$ {\left\{{\lambda }_{i}\right\}}_{i=1}^{p} $$ of the covariance matrix chosen to keep the order $$ {\lambda }_{1}\ge {\lambda }_{2}\ge \cdots \ge {\lambda }_{p}$$. The vector components $$ w$$ are the corresponding eigenvectors of the covariance matrix. The data points in the space of the first $$ k$$ directions represent a sample in lower dimensional space that preserves the maximum information value in terms of Fisher information.

Unfortunately, it should be remembered that the PCA transformation is a linear transformation and is not optimal when the data distribution deviates from the normal distribution [31]. Therefore, often data visualization using a PCA transformation does not reveal all the interesting relationships in the data.

The calculations were done in SAS/Stat ver. 15.1, Python/PHATE ver. 1.0.9 and R/archetypes ver. 2.2 − 0.1.

## Data and encoding process

Data collected in the study includes responses from 142 pregnant women. Among the questions 17 of them related directly to physical activity were divided into 3 domains of items based on the expert knowledge.

The first domain of 6 statements (Domain 1, General Physical Activity, GPA) concerned general knowledge about physical activity and constituted a 5-point Likert scale (from “strongly agree” to “strongly disagree”). The items in this group are:

- Regular physical activity is recommended during pregnancy (GPA1).
- Regular physical activity has a positive effect on the course of pregnancy (GPA2).
- A pregnant woman doesn’t have to limit her physical activity if she does not feel tired (GPA3).
- An inactive pregnant woman can start exercising (GPA4).
- A woman who was physically active before pregnancy can continue her exercise program during pregnancy (GPA5).
- Home activities can replace additional exercise (GPA6).

The next group of questions (Domain 2, Recommended Physical Activity, RPA) were the statements regarding the knowledge about physical activities that are allowed in pregnancy. This domain included 6 statements measured on a bipolar, 3-point scale (yes - right answer, I don’t know, no).

- A healthy pregnant woman should exercise several times a week (RPA1).
- A woman with an uncomplicated pregnancy can continue physical activity with the perceived exertion at a moderate level (RPA2).
- A woman with an uncomplicated pregnancy can lift weights of 3–5 kg (RPA3).
- Allowed in uncomplicated pregnancy: low impact aerobic (RPA4).
- Allowed in uncomplicated pregnancy: jogging (RPA5).
- Allowed in uncomplicated pregnancy: swimming (RPA6).

The last group (Domain 3, Non recommended Physical Activity, NPA) consisted of 5 statements and concerned the knowledge about not recommended physical activities, such as lifting heavy weights, continuing too much effort, outdoor cycling, skating/roller skating. These questions, like the questions from group 2, were constructed on a bipolar, 3-point scale (yes-right answer, I don’t know, no - wrong answer).

- A healthy pregnant woman doesn’t have to exercise every day (NPA1).
- A pregnant woman should discontinue physical activity when she feels exertion as hard (NPA2).
- A pregnant woman should not lift heavy items (10 kg or more) (NPA3).
- Not recommended in pregnancy: riding an outdoor bike (NPA4).
- Not recommended in pregnancy: skates /rollerblades (NPA5).

The coding process was as follows. For statements from Domain 1, correct knowledge is rewarded with the highest number of points (5), and an incorrect answer gives 1 point.

For the statements from Domains 2 and 3, correct answers regarding permitted or not recommended activities during pregnancy were assigned values of 2, incorrect 0, and the neutral answer “I don’t know” was marked with 1.

Thus, each respondent is characterized on the one hand by 17 values, which are points assigned to the answers to 17 questions divided into 3 domains, and on the other hand, by 3 values (scores) which are the sums of the points obtained from the answers to the questions in each of the 3 domains separately.

Range of the score for the items in Domain 1 was 6–30, in Domain 2: 0–12 and in Domain 3: 0–10.

All variables were normalized^2^ so that they could be treated as equally important. For a variable ${x}_{j}$ whose high values are desired, transformed variable is created according to the formula:

$$ {z}_{ij}=\frac{{x}_{ij}-{\underset{i}{\text{min}}({x}_{ij})}_{ }}{\underset{i}{\text{max}}({x}_{ij}) -\underset{i}{\text{min}}\left({x}_{ij}\right)}.$$

New variables take values in the interval $$ \left[\text{0,1}\right]$$. If $$ {x}_{ij}=max{(x}_{ij})$$, then $$ {z}_{ij}=1,$$ and if $$ {x}_{ij}=\text{m}\text{i}\text{n}\left({x}_{ij}\right)$$, then $$ {z}_{ij}=0. $$ For variables in Domain 1 $$ max{(x}_{ij})=5$$, and $$ min{(x}_{ij})=1.$$ For variables in Domains 2 and 3 $$ \text{m}\text{a}\text{x}\left({x}_{ij}\right)=2$$, and $$ min{(x}_{ij})=0$$.

As a result of this transformation the range of points for each question was [0,1] so that all questions were given the same weight. The value 0.5 for a separate question indicates lack of knowledge (“I don’t know”). Values below 0.5 indicate wrong knowledge, while above 0.5 a good one. Maximal total scores for questions in sequential domains (GPA, RPA, NPA) were 6, 6 and 5, respectively. The maximal total score was 17.

The minimum in the analysed data was 8.03 (47% of 17), and maximum was 16.25 (96% of 17). This means that there are no women with incorrect knowledge in all areas. There is also no woman with perfect knowledge in all areas.

## Results

Archetypal analysis was performed based on unified values. An expert method was used to choose a model with $$ k=3$$ archetypes.^3^ The calculations were made using the Archetypes package in the R language. The algorithm uses the Monte Carlo technique, so in order to obtain stable results, 5000 repetitions were made. Three weights were calculated for each observation. The weights are the coefficients of combinations of archetypes for each woman and can be regarded as percentage participation of archetypes. Values close to 1 indicate that the observation is related to the archetype in question.

**Fig. 1:**
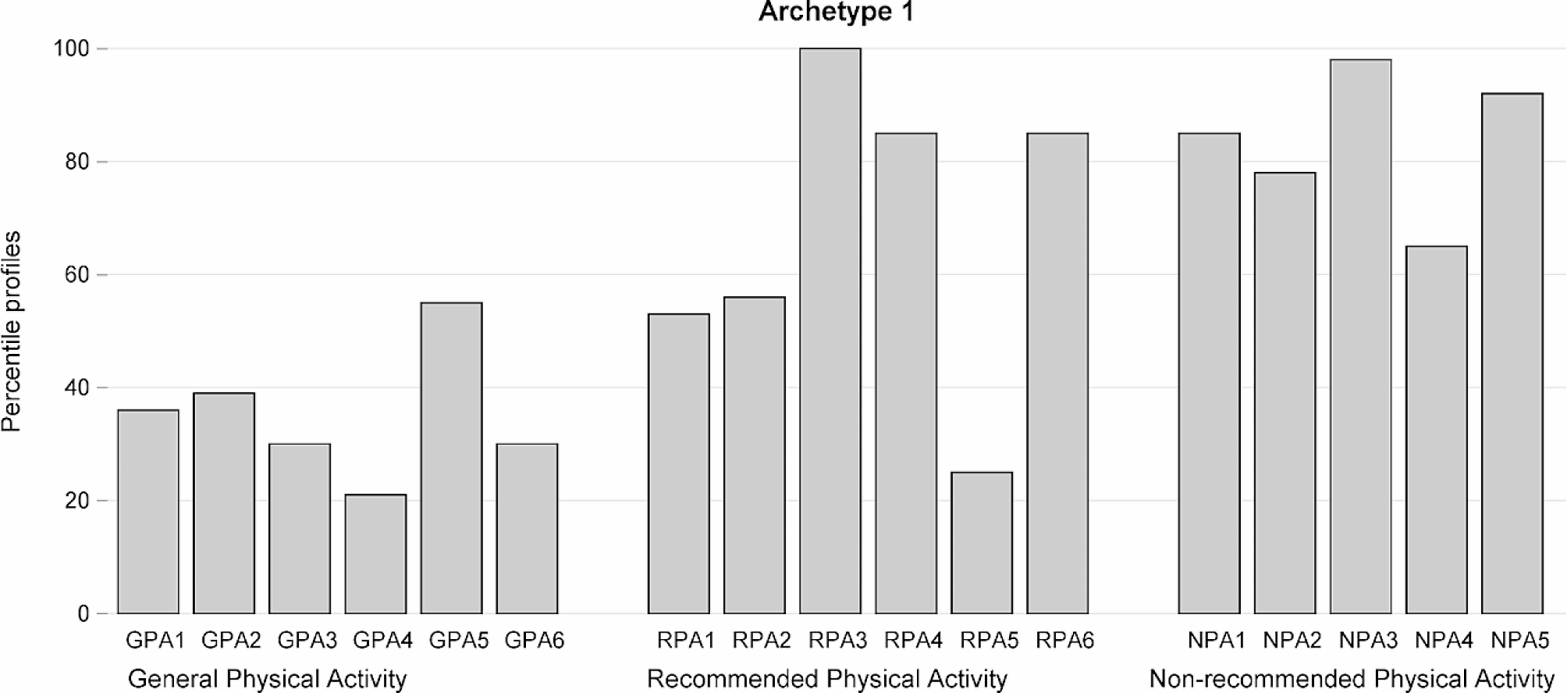
Answers of a “theoretical” woman representing Archetype 1 expressed as proportion of the maximum standardized value for each of 17 questions divided into 3 domains

Archetype 1 (Fig. 1) represents a woman who has incorrect general knowledge, chaotic knowledge of permitted activities and correct knowledge about not recommended activities. The total result this woman would obtain is 10.2 (60% of 17). The woman representing this archetype would get hardly 2.06 points (34% of 6) for general knowledge, which is little. She would get 3.98 points (66% of 6) for questions examining her knowledge of recommended activities and 4.16 points (83% of 5) for questions representing knowledge of not recommended activities, which is much. The woman representing this archetype could be described as a cautious woman. Table 1 shows totals of points gathered in three domains of questions and their sum for women closely related to Archetype 1. There are only 5 observations with weights at least 0.8 for that archetype. One woman matches exactly that archetype and this is the woman with the lowest total in the first group of questions. Similarly, all women close to that archetype have low scores for questions in Domain 1 (average total is 2.18, (36% of 6)) what confirms incorrect general knowledge and is close to the result of the archetypal woman. The average total for questions in Domain 3 is 4 (80% of 5) which confirms good knowledge of not recommended activities and which is close to the score of the archetype.

**Table 1:** Normalized scores of women close to Archetype 1 (weights at least 0.8)

ID	Total	Sum Domain 1 GPA	Sum Domain 2 RPA	Sum Domain 3 NPA	Archetype 1 Weight
64	9.58	3.08	3.50	3.00	0.91
102	9.08	2.08	4.00	3.00	0.98
113	11.00	1.00	5.00	5.00	1.00
143	8.08	2.08	2.00	4.00	0.99
144	10.67	2.67	3.00	5.00	0.86
Average	9.68(57%)	2.18(36%)	3.50(58%)	4.00(80%)	

**Fig. 2:**
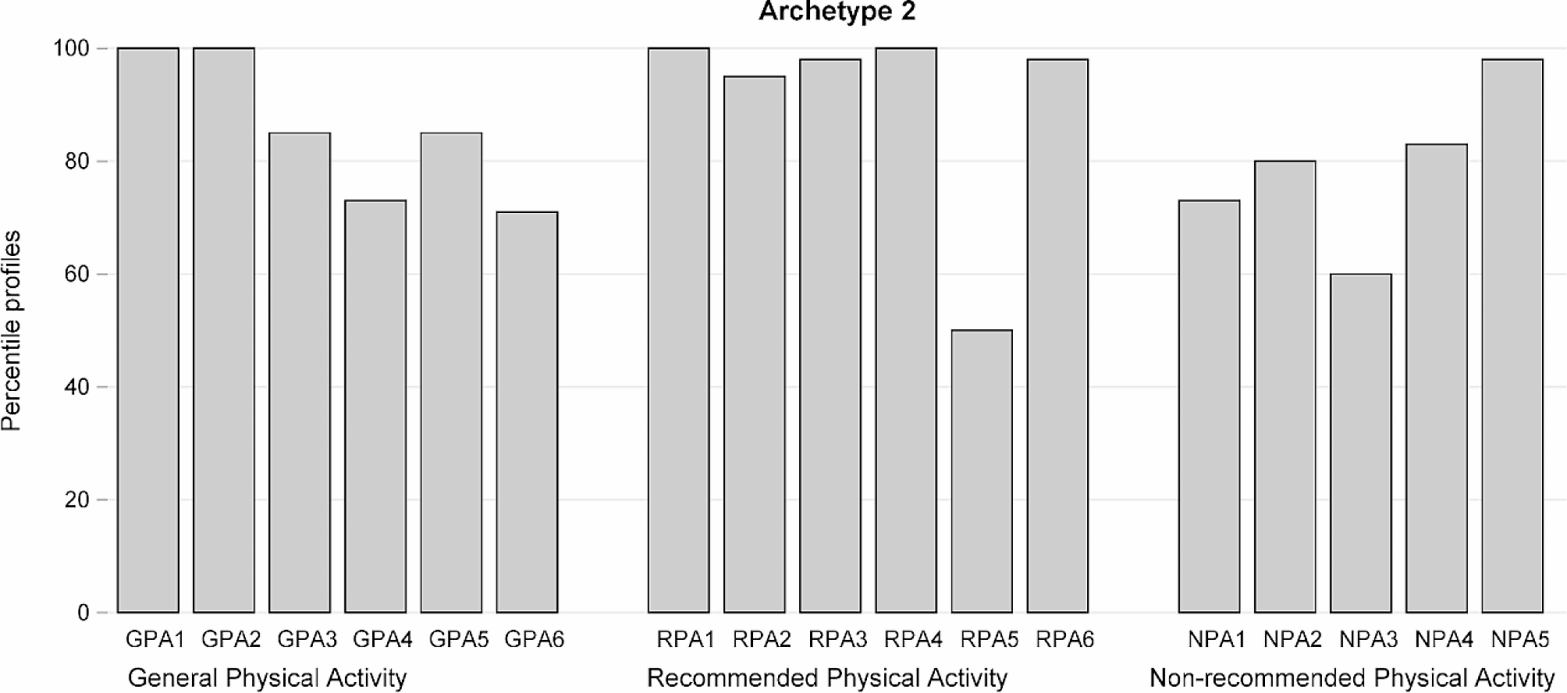
Answers of a “theoretical” woman representing Archetype 2 expressed as proportion of the maximum standardized value for each of 17 questions divided into 3 domains

Archetype 2 represents a woman with relatively good knowledge in all three areas (Fig. 2). The total score an archetypal woman would get for all questions is 14.39 (85% of 17). She would get 5.07 points (85% of 6) and 5.41 points (90% of 6) for questions in Domain 1 and 2 respectively, and 3.9 points (78% of 5) for questions representing knowledge of not recommended activities. The woman representing this archetype could be described as a well informed and aware person.

**Table 2:** Normalized scores of women close to Archetype 2 (weights at least 0.8)

ID	Total	Sum Domain 1 GPA	Sum Domain 2 RPA	Sum Domain 3 NPA	Archetype 2 Weight
15	14.50	4.50	6.00	4.00	0.89
19	15.00	5.00	5.00	5.00	0.89
20	15.00	6.00	4.50	4.50	0.85
25	11.75	4.25	6.00	1.50	0.85
34	14.00	5.00	5.00	4.00	0.96
37	14.50	4.50	5.00	5.00	0.82
48	15.25	5.25	5.00	5.00	0.93
54	13.50	5.50	5.50	2.50	0.94
57	14.00	5.00	4.00	5.00	0.80
59	14.00	5.00	6.00	3.00	0.84
66	15.75	5.75	6.00	4.00	0.93
73	14.25	5.25	6.00	3.00	0.97
90	14.75	5.75	5.00	4.00	0.97
91	13.25	5.75	5.50	2.00	0.97
114	13.25	5.25	5.00	3.00	0.84
116	14.50	5.50	5.00	4.00	0.82
118	16.25	5.25	6.00	5.00	0.85
123	13.25	5.25	6.00	2.00	0.84
128	15.00	5.00	5.00	5.00	0.81
129	12.50	4.50	5.00	3.00	0.88
132	15.00	5.50	4.50	5.00	0.92
138	14.75	4.75	6.00	4.00	0.90
150	15.25	5.25	5.00	5.00	0.92
Average	14.32 (88%)	5.16 (86%)	5.30 (88%)	3.85 (77%)	

There are 23 women close to Archetype 2 (Table 2). The average of the total is 14.32 (88% of 17) which confirms relatively good knowledge in all 3 areas. The average total scores for questions in Domains 1 and 2 are very high, 5.16 (86% of 6) and 5.30 (88% of 6) respectively, which is close to the result of the Archetype 2. The average for questions in Domain 3 is 3.85 (77% of 5), which again is close to the result of the Archetype 2.

**Fig. 3:**
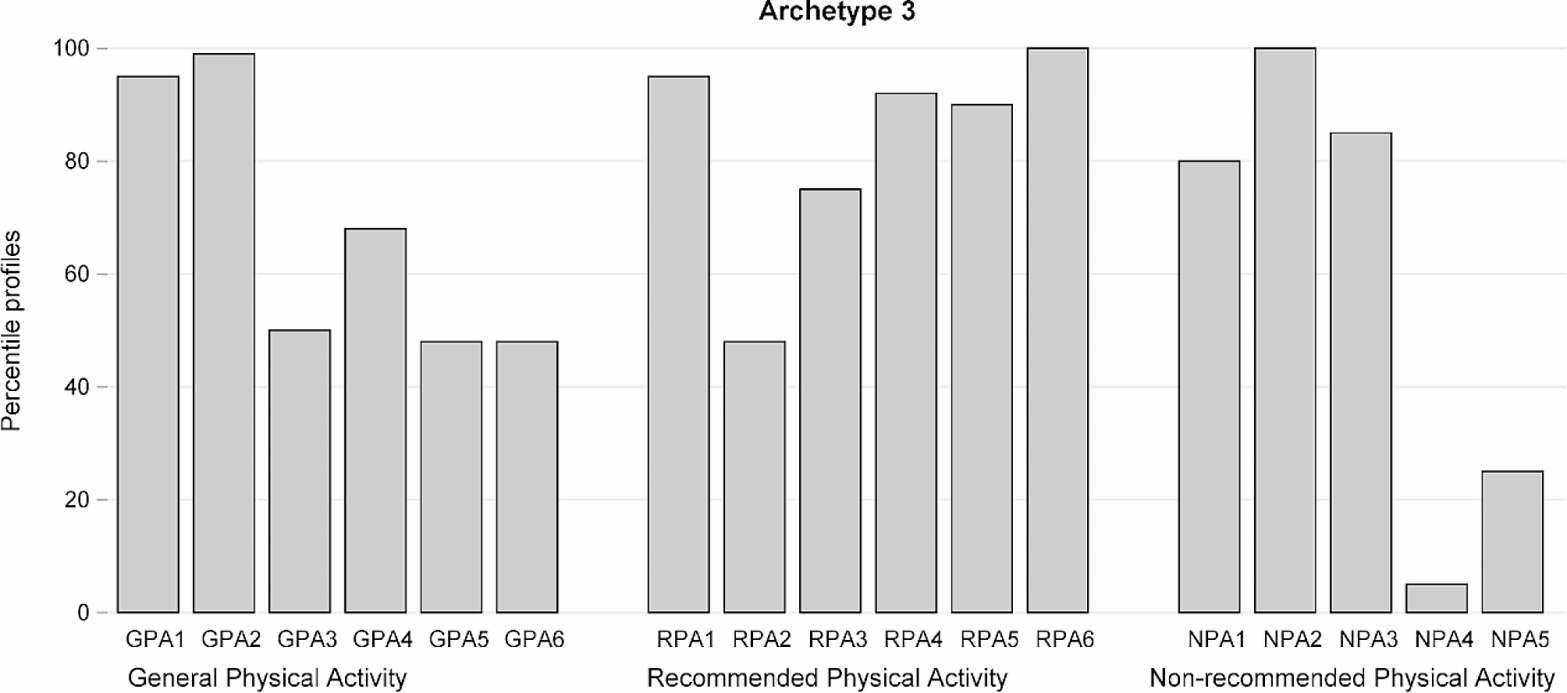
Answers of a “theoretical” woman representing Archetype 3 expressed as proportion of the maximum standardized value for each of 17 questions divided into 3 domains

Archetype 3 (Fig. 3) represents a woman with relatively good general knowledge and relatively good knowledge of recommended activities, and chaotic knowledge of not recommended activities. This woman gives correct answers to questions in Domain 1 and 2 or admits lack of knowledge for these questions and gives both correct and incorrect answers for questions in Domain 3. The total of points this woman would obtain is on average 12.1 (71% of 17). The women representing this archetype would get 4.09 points (68% of 6) for general knowledge. She would get 5.13 points (86% of 6) for questions examining her knowledge of recommended activities which is really much and 2.89 points (58% of 5) for questions representing knowledge of not recommended activities, which is little. She could be described as an unaware woman who thinks any activity is allowed.

There are 6 observations with weights at least 0.8 close to Archetype 3. Table 3 shows totals of points gathered in three domains of questions and their sum for women close to Archetype 3.

**Table 3:** Normalized scores of women close to Archetype 3 (weights at least 0.8)

ID	Total	Sum Domain 1 GPA	Sum Domain 2 RPA	Sum Domain 3 NPA	Archetype 3 Weight
17	12.5	4.00	5.50	3.00	0.86
22	10.0	3.00	5.00	2.00	0.81
26	11.8	4.25	4.00	3.50	0.96
53	11.4	3.42	6.00	2.00	0.82
101	12.5	4.50	5.00	3.00	0.90
117	12.5	4.50	5.50	2.50	0.95
Average	11.8(73%)	3.9 (66%)	5.2(86%)	2.7(53%)	

The total scores for women close to archetype 3 range from 10.0 to 12.5. The total score for questions in Domain 1 is not less than 3 with average 3.9 (66%). The total score for questions in Domain 2 is not less than 4, and the average is 5.2 (86% of 6), very close to the result of the Archetype 3. On other hand the total score for questions in Domain 3 is low, not exceeding 3.5, with average 2.7 (53% of 5), also close to the result of the archetypal woman.

**Fig. 4:**
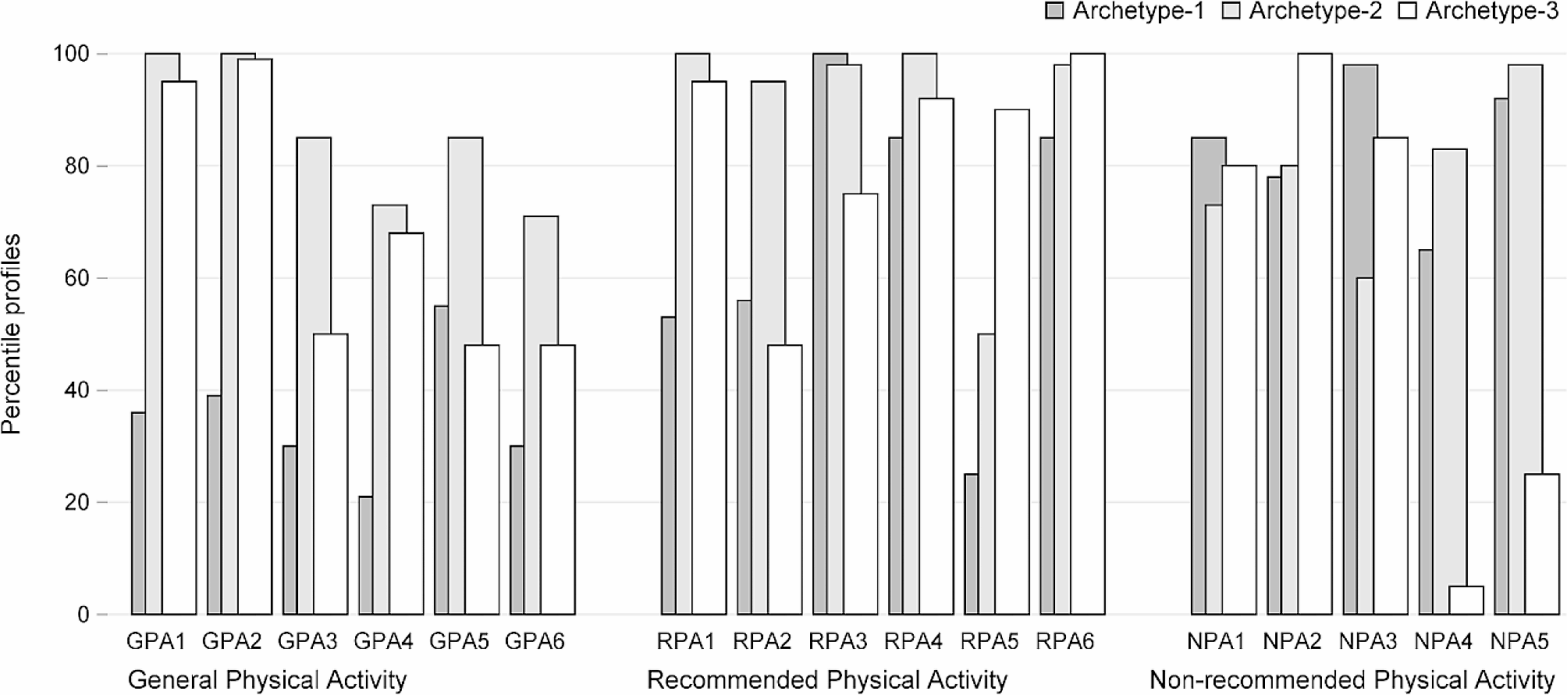
Comparison of archetypes– scores for 17 questions divided into 3 domains

Figure 4 shows comparison of archetypes. The archetypes well distinguish knowledge depicted by separate questions. The differences between them are reflected by totals of points for each group of questions and their sums (Table 4).

**Table 4:** Total values for questions in separate domains and the sum obtained by archetypal woman as well as average, minimal and maximal values

	Total	Total Domain 1 GPA	Total Domain 2 RPA	Total Domain 3 NPA
Archetype 1	10.20(60%)	2.06(34%)	3.98(66%)	4.16(83%)
Archetype 2	14.39(85%)	5.07(85%)	5.41(90%)	3.90(78%)
Archetype 3	12.10(71%)	4.09(68%)	5.13(86%)	2.89(58%)
Mean total	12.66(74%)	3.92(65%)	4.97(83%)	3.76(75%)
Min total	8.08(47%)	1.00(17%)	2(33%)	1.5(30%)
Max total	16.25(96%)	6.00(100%)	6(100%)	5(100%)

**Fig. 5:**
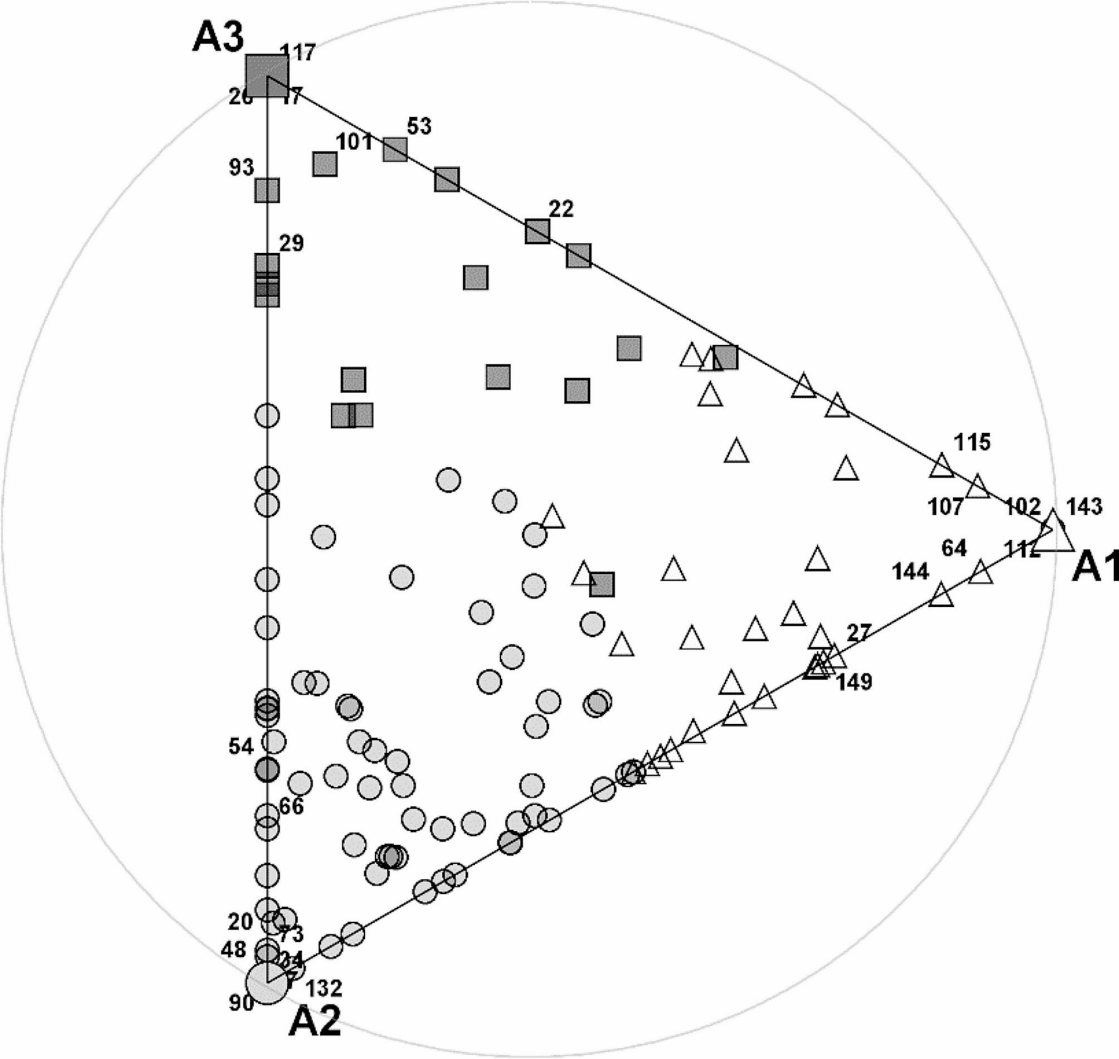
Visualization of observations as convex combinations of archetypes. The numbers are IDs of previously considered women, the most representative for archetypes. A1, A2, A3 denote relevant archetypes. Objects close to Archetype 1 are marked as triangles. Objects close to Archetype 2 are marked as circles and objects close to Archetype 3 are marked as squares

The featured archetypes provide insight into general tendencies in levels of knowledge of investigated women. Archetypes are not only extreme observations but representatives of certain groups of objects that can be observed in Fig. 5. It shows individuals in the space spanned by archetypes. There are many objects close to Archetype 2 (circles). It could indicate that the knowledge represented by the related archetypal woman is somehow representative to the majority of investigated women. The other archetypes are not so abundantly represented.

**Fig. 6:**
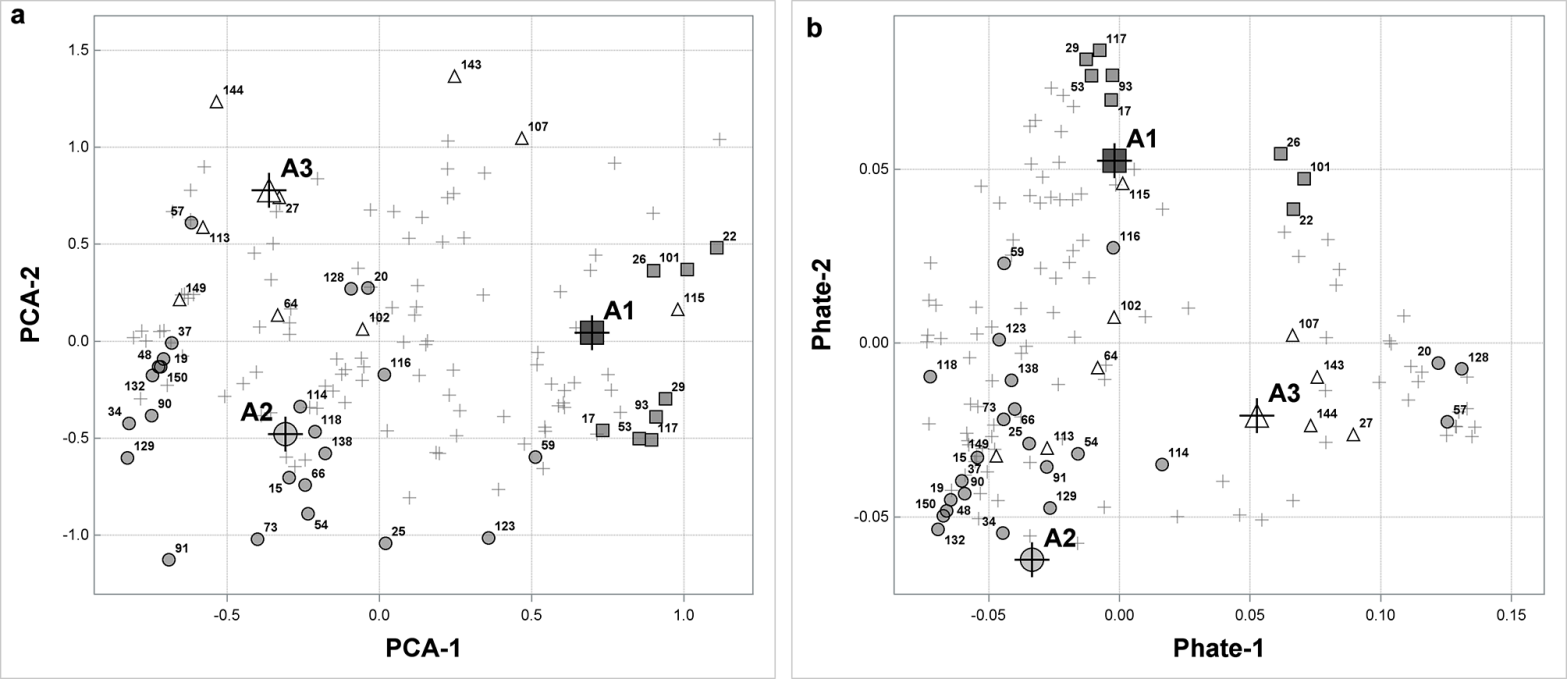
Representation of archetypes against data. Left (**a**): in the space spanned by first two principal components; Right (**b**): Visualization using PHATE in the space spanned by two first PHATE factors. IDs of previously considered women, the most representative for archetypes are given. Objects close to Archetype 1 are marked as triangles. Objects close to Archetype 2 are marked as circles and objects close to Archetype 3 are marked as squares

Our objective is to discover patterns of knowledge. Therefore, we visualize our raw data in a lower dimensional space to see how the observations are related to each other and to archetypes. Namely, we present archetypes and raw data in a two-dimensional space spanned by the first two principal components (Fig. 6a) which is a traditional approach to that problem and in space spanned by PHATE coordinates (Fig. 6b) which is a relatively new visualization method.

As in Fig. 5, archetypes in Fig. 6a are extreme observations. Moreover, the only concentration of objects is close to Archetype 2 (A2). We do not observe any other clusters of objects.

A more complex structure of data is presented in Fig. 6b. The picture was obtained with PHATE algorithm and it shows a more dispersed, nonlinear structure of data. Apart from Archetype 2, the other archetypes do not represent extreme observations in the data space (A1 is in the middle of the set). Moreover, there are some observations that although close to Archetype 2, are located in a separate island away from it.

Detailed analysis revealed that formation of separate subgroups externally to the archetype positions was conditioned by the answers to particular questions (Fig. 6b).

Women in Fig. 6b with coordinate Phate 1 greater or equal 0.1, although with weights assigning them to different archetypes, have similar level of knowledge of recommended activities (Domain 2). The average in this set is 4.36 (73% of 6) which is higher than for Archetype 1 but lower than for Archetypes 2 and 3. On the other hand the average total for activities that are not advised (Domain 3) is 4.56 (91% of 5) and it is higher than the values for all distinguished archetypes. That means that women in this set have common and good knowledge of not recommended activities. They are perfectly consistent and correct as far as questions NPA1, NPA2 and NPA3 are concerned. The only question where various answers were given is NPA4 (riding a bike). The average total value in the examined set for Domain 1 (GPA) is 4.14 (69%) and it is higher than the values obtained by Archetype 1 and 3. The women in this set gave correct answers or admitted lack of knowledge for questions GPA1, GPA2 and GPA3.

We can also have a deeper look into the set of objects in the left bottom corner of Fig. 6b. We have examined objects that have coordinate Phate 1 below − 0.04 and Phate 2 below − 0.025 that corresponds to that set. The women in this set have similar knowledge of recommended activities. The knowledge is correct but for jogging (RPA5). For this question the majority of women in the investigated set gave incorrect answer. The average total for questions in Domain 2 is 5.17 (86% of 6) which is high. The women in this set have relatively good knowledge of not recommended activities with the average total 4.33 (87% of 5).

We can notice that PHATE allowed for distinguishing two larger sets of objects. One is left to Archetype 1 (A1), the other right to it. The women in the left set, with Phate 1 less or equal 0.02 have all but five given correct answer to question RPA2, while the women in the set right to A1, with Phate 1 greater than 0.05, gave incorrect answer to that question. There are no visible differences in answers to other questions. These findings are justified substantively and will be described in more detail in the next paper.

We have examined objects that have coordinate Phate 1 below 0 and Phate 2 above 0.04 which corresponds to elements in the upper left corner in Fig. 6b. These women have common and correct knowledge of recommended activities. The average total for questions in Domain 2 is 5.57 (93% of 6) which is very high and higher than for any archetype. The women in the set have common and correct knowledge of not recommended activities as far as questions NPA1 and NPA2 are concerned and incorrect knowledge as far as question NPA4 (riding a bike) is concerned. The average total for questions in Domain 3 is 3.3 (66% of 5) which is low.

In all distinguished sets women have similar and chaotic general knowledge.

PHATE allowed for distinguishing clusters of objects that have common features but were not captured by Archetypal analysis (also in the case where more archetypes were taken into account). The analysis we have performed indicates that PHATE gives a deeper insight into the data than PCA and captures its nonlinear structure.

Archetypal analysis allows to determine the structure of the base patterns representing the state of knowledge of women. The dominant pattern is represented by Archetype 2. This means that, according to the data collected and presented in Fig. 5 most women have relatively good knowledge in all of the areas studied. In fact, 97 women (68%) scored over 50% in all three domains. However, the research showed that majority of pregnant women have chaotic general knowledge (GPA). On the other hand, investigated group of women represented good knowledge of recommended activities. The only question that received many wrong answers (34%) was that about jogging (RPA5). This group of women is represented by Archetype 1. 92% women assigned to that archetype admitted no knowledge or had wrong knowledge in that area. 97% of women assigned to Archetype 3 admitted no knowledge or had wrong knowledge concerning riding an outdoor bike in pregnancy (NPA4). This can be caused by the fact that riding a bike is a popular recreational activity but it also serves as a means of transport. It has to be stressed that the differentiating questions RPA5 and NPA4 were captured only just by PHATE.

## Discussion

Our goal was to select patterns of knowledge and to score total knowledge of pregnant women on physical activity. Our novel idea was to use simultaneously Archetypal analysis and PHATE for getting a deep insight into the data structure.

Distinguishing archetypes allowed to determine the base patterns representing the state of knowledge of women. Traditional pattern analyses come to the use of dimensionality reduction or segmentation methods - the focus is on exploration the homogeneous groups. In marketing research, a different approach is often used, based on the so-called trend-makers - extreme patterns that allow to define “clean” objects. Archetype analysis, that we have applied, goes in this direction. Apart from the identification of the archetypes, the graphic presentation proved to be very helpful in the interpretation of the results. For this, a reduction of dimensionality is recommended. This article compares the traditional PCA method used for dimensionality reduction and the more complicated but also intuitive algorithm PHATE. The analysis revealed differences between the space presentation by linear PCA projections and the non-linear PHATE representation. The graphic maps only partially overlap. In the case of clear monotonic relationships, which are reflected in strong dose-response effects, PCA visualization is a very good tool. The situation is different in the case of more subtle, nonlinear dependencies. In our case, it seems reasonable to assume non-monotonic nature of the relationship between the responses to the questionnaire and the state of knowledge. This suggests the existence of more sophisticated relations that distort the simple monotonic “dose-response” image and may result from chaotic knowledge. In our opinion, the presentation of the results in the PHATE space supplements interpretation of the archetypes.

## Conclusions

The goal of the research was to select patterns of knowledge and to score total knowledge of pregnant women on physical activity. Our novel idea, combining Archetypal analysis with PHATE, proved to be a highly efficient tool in examining the structure of knowledge reflected by answers in the survey. Thanks to that approach we were able to determine and describe groups of women with similar levels of knowledge in a deeper way than it was possible using PCA.

The methods we have chosen allowed us to distinguish patterns of pregnant women knowledge on physical activity preserving full dimensionality of questions used in the questionnaire. We were able to distinguish groups of women with a similar range of knowledge and to identify areas where knowledge is incomplete.

In the next step we intend to verify described here methods on the new set of Polish women at the beginning of pregnancy, study socio-demographic characteristics of women in the context of distinguished archetypes and analyze changes of the patterns of knowledge during pregnancy.

The results can be helpful in determining public health courses of action and methods aiming to promote physical activity of pregnant women.

### Electronic supplementary material

Below is the link to the electronic supplementary material.

Supplementary Material 1

## Data Availability

Data that supported the findings of this study is a property of the Institute of Mother and Child in Warsaw.

## Footnotes

1 All pregnant persons identified as women.

2 R-archetypes algorithm adds its own automatic standardization (mean/std). We have used (min/max) normalization to make variables equally important and to create variables used to identify archetypes.

3 We have also performed models with k = 4. Trials with k = 4 gave a very similar RSS goodness-of-fit statistic value as for k = 3. Identification of archetypes for k = 3 was more suitable for interpretations.

## Abbreviations

PA: Physical Activity
PCA: Principal Component Analysis
PHATE: Potential of Heat-diffusion for Affinity-based Trajectory Embedding
GPA: General Physical Activity
RPA: Recommended Physical Activity
NPA: Non recommended Physical Activity
