# Supplementary material for: Archetype analysis and the PHATE algorithm as methods to describe and visualize pregnant women’s levels of physical activity knowledge
Source: BMC Public Health. 2024 Apr 15;24:1054. doi: 10.1186/s12889-024-18355-7 (PMC11020919; doi:10.1186/s12889-024-18355-7)
Supplement: Supplementary file 1 — Supplementary Material 1 [file 12889_2024_18355_MOESM1_ESM.docx]

**
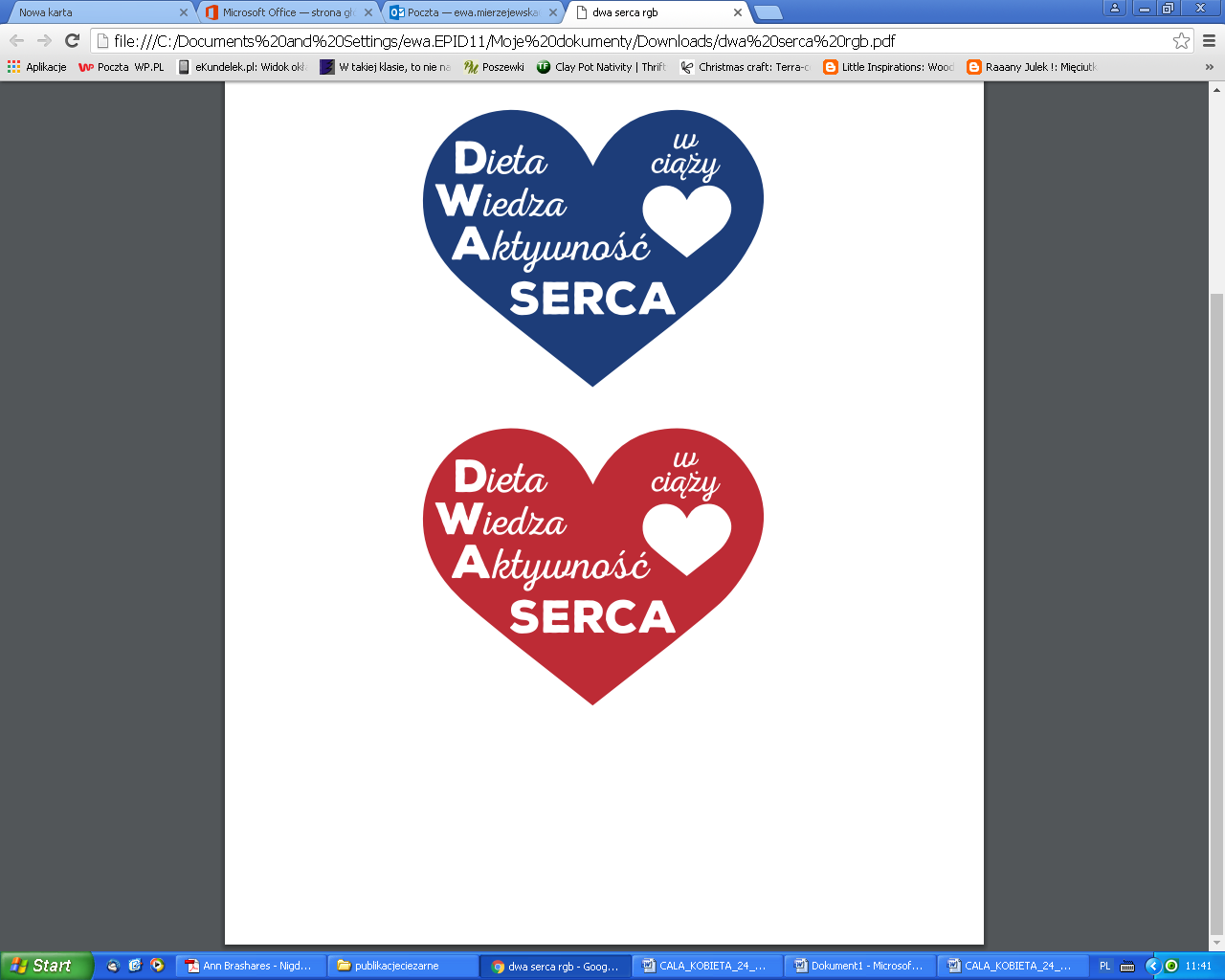
KNOWLEDGE ABOUT PHYSICAL ACTIVITY – QUESTIONNAIRE**

| **Do you agree with the following statements?** | Strongly agree | Agree | Neither agree or disagree | Disagree | Strongly disagree |
| --- | --- | --- | --- | --- | --- |
| 1. Regular physical activity during pregnancy is indicated |  |  |  |  |  |
| 1. Regular physical activity during pregnancy has a positive effect on the course of the pregnancy |  |  |  |  |  |
| 1. A pregnant woman should organize her physical activity herself, even if she feels tired |  |  |  |  |  |
| 1. A woman who was not physically active (had a sedentary lifestyle) prior to her pregnancy should not begin to exercise during pregnancy |  |  |  |  |  |
| 1. A woman who was physically active prior to pregnancy can continue her physical activities during pregnancy |  |  |  |  |  |
| 1. Physical activity associated with household chores can replace additional exercises |  |  |  |  |  |

7. A woman with a normal pregnancy should exercise:

- Everyday
- Few times a week
- Once a week
- Not at all
- I don’t know

8. W During physical activity, a woman with a normal pregnancy should stop exertion when it feels:

- Light
- Moderately heavy
- Heavy
- Very heavy
- I don’t know

9. A woman with a normal pregnancy should not carry weights heavier than:

- 1 kg
- 3 kg
- 5 kg
- 10 kg
- I don’t know

**Are the physical activity types below allowed for women with a normal pregnancy?**

|  | **Yes** | **No** | **I don’t know** |
| --- | --- | --- | --- |
| 1. Low impact prenatal aerobics |  |  |  |
| 1. Jogging |  |  |  |
| 1. Riding a bicycle |  |  |  |
| 1. Riding roller skates |  |  |  |
| 1. Swimming |  |  |  |

**Domains**:

1. General Physical Activity: questions 1, 2, 3, 4, 5, 6
2. Recommended Physical Activity: questions 7, 8, 9, 10. 11, 14
3. Non-recommended Physical Activity: questions 7, 8, 9, 12, 13
